# Understanding a Complex Intervention to Reduce Unplanned Hospitalizations From Nursing Homes: Process Evaluation of INTERCARE

**DOI:** 10.1002/hsr2.71748

**Published:** 2026-01-11

**Authors:** Franziska Zúñiga, Kornelia Kotkowski, Raphaëlle Ashley Guerbaai, Michael Simon, Christine Serdaly, Sabina De Geest, Andreas Zeller, Reto W. Kressig, Nathalie I. H. Wellens, Thekla Brunkert

**Affiliations:** ^1^ Nursing Science (INS), Department Public Health (DPH), Faculty of Medicine University of Basel Basel Switzerland; ^2^ Rehabilitation, Ageing, and Independent Living (RAIL) Research Centre, School of Primary and Allied Health Care Monash University Frankston Victoria Australia; ^3^ Serdaly and Ankers snc, Conches Switzerland; ^4^ Department of Public Health and Primary Care, Academic Centre for Nursing and Midwifery KU Leuven Leuven Belgium; ^5^ Centre for Primary Health Care University of Basel Basel Switzerland; ^6^ Faculty of Medicine Basel University of Basel Switzerland; ^7^ University Department of Geriatric Medicine FELIX PLATTER Basel Switzerland; ^8^ La Source School of Nursing HES‐SO University of Applied Sciences and Arts of Western Switzerland Lausanne Switzerland; ^9^ Faculty of Health Sciences and Medicine University of Lucerne Switzerland

**Keywords:** complex intervention, hospitalization, long‐term care, nursing home, process evaluation

## Abstract

**Background and Aims:**

The INTERCARE study aimed to reduce unplanned hospitalizations in Swiss nursing homes (NH) by implementing a complex nurse‐led model featuring nurses with in‐depth geriatric training (INTERCARE nurse). To optimize the intervention for scale‐up, this process evaluation aims to describe the intervention elements' use in practice, to explore related barriers and facilitators, and to explore the mechanisms of change.

**Methods:**

The process evaluation was conducted alongside the INTERCARE study guided by the Medical Research Council's framework. We combined qualitative data from participating NHs via individual interviews and focus groups with care workers, INTERCARE nurses and physicians. Additionally, structured notes from meetings with NH leadership teams were included. For each intervention element, data were compiled into a spreadsheet, followed by inductive coding. Changes reported by NHs related to the intervention were summarized and consolidated in a conceptual model.

**Results:**

Our analysis revealed two groups of intervention mechanisms: (1) those changing care workers' reasoning, for example, following a more structured approach to perform tasks (e.g., with the help of evidence‐based instruments) and (2) those enabling existing resources, for example, availability of a responsible contact person (i.e., the INTERCARE nurse). We further identified behavior changes in care workers, including faster reaction to changes in resident situations, more comprehensive assessment of resident situations and improved communication, which contributed to the reduction of hospitalizations.

**Conclusions:**

Understanding how interventions work in practice is crucial to inform potential adaptations to complex interventions. We found interprofessional collaboration supported by evidence‐based instruments and the INTERCARE nurse to be key elements to drive the reduction of unplanned hospitalization, together with the use of ACP. The findings can help not only to optimize the intervention elements for scale‐up, but also hold implications for refining alternative care models, ultimately reducing unplanned hospitalizations in NH residents.

**Trial Registration:** ClinicalTrials.gov identifier: NCT03590470.

## Summary

- The INTERCARE intervention reduced hospitalization rates and was well‐received by nursing homes.
- Reviewing individual intervention elements of INTERCARE showed essential elements to produce change in unplanned transfers.
- Process evaluations reveal how complex interventions work in practice, guiding adaptations to increase effectiveness and feasibility.

## 1 Introduction

Unplanned hospitalizations of nursing home (NH) residents can lead to severe complications such as infections, falls, or delirium, significantly impacting their quality of life [1, 2]. A multitude of interrelated and complex factors contribute to unplanned hospital admissions. These can include the absence of advanced care directives among NH residents, lack of clarification of resident and family preferences, staff's inability to recognize early symptoms of deteriorating health conditions, and suboptimal communication between hospitals and NHs [3].

Previous initiatives have demonstrated mixed effectiveness in reducing hospitalizations of NH residents [4, 5, 6]. These are often complex interventions combining multiple elements, drawing on novel models of care. Despite their complexity in targeting multiple levels within a system and involving various groups or individuals, most of these interventions lack comprehensive evaluations of their individual elements. This can impede a clear understanding of how each element contributes to the intervention's overall effectiveness.

Process evaluations seek to scrutinize the mechanisms to better understand how complex interventions affect change and identify the contextual factors influencing outcomes [7, 8]. Such evaluations are recommended by the Medical Research Council's framework for complex intervention research, using qualitative or mixed‐method design to provide answers about the fidelity and quality of an implementation, the mechanisms of change, and the impact of context [8]. This is crucial to optimizing intervention elements and implementation strategies to prepare interventions for scale‐up.

There is limited literature that specifically reports on process evaluations of complex interventions such as nurse‐led models of care implemented in NHs to reduce hospital transfers. The Improving INTERprofessional CARE for Better Resident Outcomes: INTERCARE is a multi‐center implementation study that tested a complex intervention to reduce unplanned hospitalizations in Swiss NHs [9]. INTERCARE provided evidence that a carefully implemented, nurse‐led care model in nursing homes can positively impact resident outcomes, such as unplanned hospitalizations [10]. An evaluation of implementation processes and outcomes showed high uptake, acceptability, feasibility, and fidelity to the intervention/model's elements [11, 12]. Understanding the factors that influence successful implementation, as well as the effects of individual intervention elements and how they are applied, is crucial. This knowledge not only helps prioritize efforts and resources and prepare for large‐scale application (scale‐up), but also supports replicability across different countries and settings.

Consequently, this study's overarching objective is to gain an understanding of how the INTERCARE intervention contributed to the reduction in unplanned hospitalizations, as observed in the main study [10]. The specific aims were (1) to describe how the intervention elements were used in practice; (2) to explore how the intervention elements contributed to a reduction in unplanned hospitalizations by examining the underlying mechanisms of change; and (3) to explore factors that facilitated or hindered the effect of individual intervention elements.

## 2 Methods

### 2.1 Design

This is a multi‐site process evaluation using a multi‐method triangulation design that combines qualitative data from various sources. This process evaluation is part of a Hybrid Type 2 effectiveness—implementation design, using a non‐randomized stepped‐wedge trial [13].

### 2.2 Intervention

INTERCARE is a complex intervention that was developed based on several elements from the previously tested INTERACT quality improvement intervention [14]. Intervention elements were adapted to the Swiss setting based on a contextual analysis and in collaboration with a local stakeholder group including representatives of residents, NH leaders, professional organizations, and health policymakers, amongst others [9]. INTERCARE comprises six core elements (Table 2 in Section Results, 3): (1) strengthening of interprofessional collaboration; (2) an INTERCARE nurse appointed to each participating NH; (3) comprehensive geriatric assessment (CGA); (4) advance care planning (ACP) to assess and document residents' care preferences and treatment choices; (5) evidence‐based tools adapted from the INTERACT intervention, that is, STOP&WATCH (an observation tool, where the acronym stands for a list of typical symptoms healthcare assistants might observe when caring for a resident), ISBAR (a tool to prepare a structured communication between healthcare professionals when a patient's health situation changes: Identification, Situation, Background, Assessment, Recommendation) and a reflection tool capturing root‐cause analysis of unplanned hospital admissions; and (6) data‐driven quality improvement to identify areas with need for improvement. Further details about the intervention elements and minimal requirements for implementation are presented below in Table 2 and in the study protocol [9].

### 2.3 Implementation Strategies

Strategies to facilitate the uptake of the INTERCARE intervention were developed considering the contextual factors of the participating NHs [9]. Central strategies comprised preparatory and ongoing training for the INTERCARE nurses, provision of ongoing consultation for NH leadership, and audit and feedback for NHs based on hospitalization data. Further details concerning the implementation strategies are provided in Table S1.

### 2.4 Sample and Data Collection

The INTERCARE study was conducted and evaluated in 11 NHs in Switzerland's German‐speaking region. The active intervention phase lasted between 12 and 18 months, depending on NH enrollment (September 2018–February 2019). Data collection was completed in all NHs in March 2020. This study employed a purposive sample of five NHs, utilizing a maximum variation sampling approach to ensure diversity in their performance trajectories, as measured by hospitalization rates in the main trial [10]. In addition, the five cases were rich in information concerning their implementation process and diverse in their approach to applying the INTERCARE model. We did not consider their legal status or location, since these did not affect their performance in the trial.

This study uses qualitative data from five different sources: (A) notes from meetings with the NH leadership; (B) notes from phone calls with INTERCARE nurses; (C) semi‐structured interviews with INTERCARE nurses 6 and 12 months after intervention start; (D) focus groups with care workers 6 months after intervention start; (E) interviews with physicians 6–9 months after intervention start. Interview guides can be consulted in the Supporting Information: S1, interview guides G1–G5. We drew on all available study data from every party involved in discussions about implementing INTERCARE. This provided a broad perspective on the process, and enabled triangulation across multiple data sources.

During the intervention period, the research team had regular meetings with the individual NH (A) leadership teams on a bi‐monthly basis and (B) INTERCARE nurse on a bi‐weekly basis to oversee the local implementation and discuss barriers and facilitators. During these meetings, the project coordinator took structured notes (themes were structures and processes of the implementation process and the six core elements of INTERCARE), which were checked and complemented after each meeting by the first author.

Individual interviews with all INTERCARE nurses (C) were conducted by a research assistant (Registered nurse (RN) with experience in qualitative research and not involved in the trial) 6 and 12 months after enrollment. The semi‐structured interviews focused on the implementation, tasks and activities of the new role as well as on the acceptance and feasibility of the intervention elements. The interviews were audio recorded and lasted on average 51 min. Subsequently, all audio data was transcribed verbatim.

The focus groups (D) were conducted by the second author (RN, doctoral student) using a knowledge mapping approach. A research assistant (RN) who was present during the interviews drafted knowledge maps and validated interview data with participants. In each of the participating NHs, two interviews were conducted, one group including only nursing assistants and the other licensed practical nurses and RNs. A semi‐structured interview guide addressing the implementation of intervention elements was developed (i.e., the INTERCARE nurse, ISBAR, and STOP&WATCH). All interviews were audio‐recorded and lasted on average 75 min. Eight out of 10 interviews were transcribed verbatim due to financial and time restrictions. The second author extracted the information from the other two interviews by listening to the audio‐recordings and making structured notes (see Section 2.5).

Physicians (E) who were responsible for at least three residents in each NH were invited to participate in a semi‐structured interview. The interviews were conducted via telephone by the project coordinator (RN) between 6 and 9 months after the intervention start. Interviews lasted between 15 and 53 min and focused on the experiences of interprofessional collaboration with care workers and the INTERCARE nurse at the NH. All interviews were transcribed.

### 2.5 Data Analysis

All available data (A–E) from the participating NHs was read by three researchers (T.B., K.B., F.Z.). Secondly, a rapid assessment procedure was used to extract and summarize data for each of the six intervention elements in a spreadsheet, as described by Vindrola‐Padros [15]. All transcripts and audio recordings from a single NH (A–E) were coded for each intervention element using three categories: Actual use (How was the intervention element used in practice?), reported impact (Which changes were reported by implementation agents related to the usage of the intervention element?) and potential barriers and facilitators (How did contextual factors influence the intervention element's effect?). After repeating this step for each NH, the findings across NHs were summarized for each intervention element using pre‐defined categories. Throughout this process, potential concerns in the extraction and interpretation of the data and the procedures were discussed between three researchers (F.Z., K.B., T.B.) during regular meetings. The extracted data were consistent across the 5 NHs. Two researchers participated in the data collection at all 11 NHs, ensuring that no aspects discussed with participants from the non‐included NHs were overlooked. To answer the three research questions, we conducted three different analyses: (I) Data reporting actual use of intervention elements was summarized and matched with the minimal requirements defined pre‐implementation for each intervention element (cf. Table S1). Actual use and individual adaptations were summarized separately. (II) Data extracted on the self‐reported effects of intervention elements were combined into a single spreadsheet. We used iterative inductive coding to create categories based on common topics for each intervention element. The new categories were summarized into a comprehensive list to facilitate cross‐comparison and consolidation. In a final step, we combined the reported effects into a conceptual model depicting potential change mechanisms towards the reduction of unplanned hospitalizations. (III) Barriers and facilitators were analyzed separately for each intervention element.

### 2.6 Ethical Considerations

This study has been approved by the responsible cantonal ethics committee “Ethikkommission Nordwest und Zentralschweiz” (EKNZ, 2018–00501) and was registered on June 18, 2018 at clinicaltrials.gov (NCT03590470). The recruitment is completed. All interview participants provided written informed consent.

## 3 Results

Overall, this process evaluation included a subsample of five NHs for the analysis. An overview of the characteristics in comparison to the other NHs participating in the INTERCARE study is shown in Table 1.

**Table 1:** Characteristics of participating nursing homes.

	NHs not included in process evaluation (*n* = 6)	NHs included in process evaluation (*n* = 5)
	*n* (%*)*	*n* (%*)*
Legal status
Privately funded	4 (66.7)	5 (100)
Publicly funded	2 (33.3)	0
Location		
Urban	4 (66.7)	4 (75.0)
Rural	2 (33.3)	0
Suburban	0 (0)	1 (25.0)
Bed count
All long‐term beds median (IQR)	134 (119–288)	114 (88–152)
Number of beds included median (IQR)	91 (85–110)	81 (72–113)
Physician model
NHs working with primary care physicians not employed by the nursing homes	1 (16.7)	3 (60.0)
NHs working with employment/contractual arrangements with physicians	3 (50.0)	0
NHs working with mixed physician models	2 (33.3)	2 (40.0)

### 3.1 Intervention Elements: Actual Use and Additional Activities

The implementation of each intervention element, based on the pre‐defined minimum requirements, is summarized in Table 2. Analysis of the data sources showed that four to five NHs met most of the minimum requirements. Regular educational sessions planned by the INTERCARE nurse with the care staff were reported by only two NHs. However, all NHs reported that the INTERCARE nurse provided daily bedside coaching to residents.

**Table 2:** Individual operationalization of intervention elements based on minimal requirements.

Elements	Minimal requirements	NH 1	NH 3	NH 6	NH 9	NH 10	Additional activities*
Interprofessional collaboration	A structure in place (e.g.,: meetings) to facilitate interprofessional communication between at least two different professionals.	✓	✓	o	✓	✓	– INTERCARE nurse introduces him‐/herself to physician (NH 3, 9, 10) and therapists (NH 1, 3, 6, 10) – Interprofessional roundtables (NH 3) – Ward rounds with INTERCARE nurse (NH 9, 10)
INTERCARE nurse	According to the INTERCARE nurse's skills and expertise residents are assessed in acute situations, when called by a member of the care team.	✓	✓	✓	✓	✓	– Rounds on the ward/proactive involvement (NH 3,6,9,10) – Conversations with informal caregivers/family (NH 3,10) – Takes over care of complex residents (NH 10)
	The INTERCARE nurse provides coaching to care staff on daily resident bedside needs.	✓	✓	✓	✓	o	– Physician visits (NH 3, 10)/calls (NH 1,6,10) – Preparation for calls with physician (NH 9) – Monitoring of resident documentation (NH 3,9,10)
	The INTERCARE nurse plans educational sessions with care staff on a regular basis.	✓	o	o	✓	o	– Management of end‐of‐life situations (NH 6,9)
	The INTERCARE nurse drives team reflections for each reflection tool filled in.	✓	✓	✓	✓	✓	– Conducting case conferences (NH 1,3,6)
Comprehensive geriatric assessment (CGA)	The INTERCARE nurse collaborates with the leadership and/or interprofessional team to discuss which assessment instrument they work with, for each of the 5 CGA dimensions in their institution.	✓	✓	✓	✓	✓	
Advance care planning (ACP)	For every newly admitted resident, the following points must be documented in the residents' records: – Do not resuscitate order – Do not hospitalize order – Use of antibiotics	✓	✓	✓	o	✓	
	For residents in unstable condition before weekends: physician orders and care plans are clarified, by the appointed responsible person(s) in each NH.	✓	o	o	✓	✓	– Proactive planning before weekends (NH 1, 10)
Evidence‐based tools	STOP&WATCH: used by nurse assistants to inform registered nurses about changes in resident condition.	✓	✓	✓	✓	✓	– Used as structuring aid for team reports (NH 3, 10)
	STOP&WATCH: the nurse responsible should perform the adequate assessment after being given the STOP&WATCH.	✓	✓	✓	✓	✓	
	STOP&WATCH: the situation for which the STOP&WATCH tool is used, is recorded in the resident's documentation, if a change in resident situation has been recognized.	o	o	✓	o	o	
	ISBAR: used by registered nurses in communicating with physician and with the INTERCARE nurse in acute situations.	✓	✓	✓	✓	✓	– Used as Email template (NH 1,3) – Preparation of physician rounds/visits (NH 3,6,9,10)
Data driven quality improvement	Continuous data collection for all hospitalizations and emergency department visits, with exports every 3 months for SPC charts and 6 months for benchmarking.	✓	✓	✓	✓	✓	
	A member of the leadership team with or without the INTERCARE nurse should discuss the SPC charts and benchmarking reports together	o	o	o	o	o	
	A member of the leadership team and the INTERCARE nurse should meet and discuss which steps are needed to improve quality improvement.	o	o	o	o	o	

Abbreviations: ACP, advance care planning; CGA, comprehensive geriatric assessment; ISBAR, introduction, situation, background, assessment, recommendation; NH, nursing home; SPC, statistical protocol chart.

✓: the element has been implemented according to the minimal requirements.

o: the element has not been implemented according to the minimal requirements.

* These additional activities are related to the minimal requirement, but would not suffice to fulfill the minimal requirement.

The NHs reported specific modifications they deemed necessary for several intervention elements, such as the personal introduction of the INTERCARE nurse to physicians and therapists or the nurse's presence during physician visits to assist communication. The INTERCARE nurse also actively engaged with ward activities, aiding in family conversations and complex situations, and was not merely present upon request.

### 3.2 Perceived Effects of Intervention Elements

**Figure 1:**
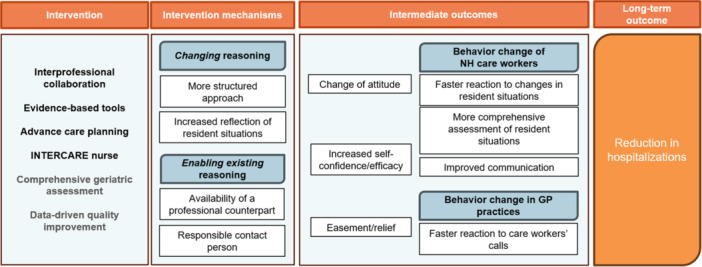
Overview of the intervention elements' mechanisms and intermediate and long‐term outcomes.

In view of aim 2, Figure 1 presents an overview of the intervention elements' mechanisms and intermediate outcomes, which include behavior modifications theorized to have resulted in fewer hospital transfers. Our analysis identified four core intervention mechanisms grouped into two categories: (I) those that aimed to change reasoning such as a “more structured approach” and “increased reflection of resident situations”, and (II) those that aimed to enhance existing reasoning through “availability of a professional counterpart“ and having a “responsible contact person”. An overview of themes and codes is provided in Table S2.

### 3.3 Mechanism: Changing Reasoning

This mechanism of change was primarily attributed to the support by the INTERCARE nurse, the employment of evidence‐based tools such as ISBAR, STOP&WATCH, the hospitalization reflection tool, and the introduction of ACP. Participants reported a more structured approach in various contexts, for instance, evaluating critical conditions of residents, preparing for physician rounds, anticipating potential exacerbations over the weekend, and facilitating team handovers.

The INTERCARE nurse served as a skilled point of contact in critical situations, coaching care workers to successfully understand and handle complex resident situations. This included the utilization of ISBAR to capture comprehensive medical history including signs and symptoms and facilitate conversations with physicians. According to the narration of the INTERCARE nurses and NH leaders, the structural aid of ISBAR coupled with successful experiences augmented care workers' self‐efficacy, their attitude towards critical situations, interprofessional collaboration, and their questioning abilities. They reported that the structured preparation of rounds or phone calls with ISBAR increased their time efficiency and productivity, leading to better‐informed physicians and improved interprofessional collaboration, including easier access when calling the GP's office.

STOP&WATCH enabled nurse assistants to be more precise during handovers, which also increased appreciation for their work. Furthermore, the hospitalization reflection tool enhanced the team's reflection of critical episodes and team learning, increasing their preparedness for future situations. The introduction of ACP raised the awareness of residents' preferences, empowering care workers to feel more confident in discussing hospitalization's pros and cons. The “more structured approach” and the “increased reflection of resident situations” were both reported as influential in changing care workers' attitudes and bolstering their self‐efficacy, resulting in behavioral modifications that decreased hospital transfers.

### 3.4 Mechanism: Enabling Existing Reasoning

The second core mechanism of change was the enabling of existing reasoning largely credited to the role of the INTERCARE nurse. INTERCARE nurses served as a coach and a counterpart in clinical discussions providing reassurance to care workers who had a designated individual to handle complex situations, thereby increasing their self‐confidence. Moreover, the responsibility assumed by the INTERCARE nurses in leading ACP discussions, and thereby helping to articulate residents' preferences, provided further relief to care teams. Their engagement in dialogues with residents and their families during challenging situations allowed care workers to focus on routine tasks, thereby alleviating stress levels.

Overall, behavior change was reported at the level of the NH care workers and the GPs. Care workers reported faster responses to changes in residents' clinical conditions, thorough assessments of situations, and enhanced communication and information flow within care teams and across professions. Similarly, GPs were seen to respond more rapidly to care workers' concerns about resident conditions, leading to a reduction in hospital transfers.

While we identified change mechanisms for four of the intervention elements—the INTERCARE nurse, interprofessional collaboration, ACP, and evidence‐based tools, the core elements of CGA and data‐driven quality improvement were rarely mentioned in interviews and did not appear to play a perceivable role in reducing hospital transfers.

### 3.5 Hindering and Facilitating Factors to Intervention Elements

Table 3 summarizes the barriers and facilitators identified from various sources for each intervention element in fulfillment of aim 3. These can be categorized into determinants related to the intervention itself, the individuals implementing it, and the NH's internal context. Lack of understanding was mentioned in relation to both CGA and data‐driven quality improvement. Participants often did not perceive the connection between these elements and the overall purpose of INTERCARE. Specifically, there was fundamental confusion about CGA's application and difficulty motivating all NH staff to engage with data.

**Table 3:** Barriers and facilitators for each intervention element.

Intervention elements	Barriers	Facilitators
Interprofessional collaboration	Limited time resources may inhibit necessary dialogue and exchange	✓ Having opportunities for exchange and relationship‐building with other professionals ✓ The presence of an open error culture facilitated learning and improvement
INTERCARE nurse	A clear understanding of the new role was not present among some care teams and ward managers, resulting in lower acceptance of the INTERCARE nurse role	✓ Shared vision between NH leadership and INTERCARE nurse ✓ Active role development with all involved parties – Participation in interprofessional meetings – Maintaining presence on the wards ✓ Exchange with INTERCARE nurses in other NHs provided opportunities for learning and sharing best practices
Comprehensive geriatric assessment	Lacking understanding of the purpose of CGA and how to make it feasible	
Advance care planning	Some facility‐specific requirements, such as conducting ACP conversations within 3 days of admission, were perceived as unrealistic INTERCARE nurses were not familiar with all residents—ACP conversations might be more effective if built on personal relationships Confidence levels in conducting ACP conversations varied among INTERCARE nurses, with some reporting low confidence The limited number of personnel trained in conducting ACP conversations restricted the availability of time resources Support from GPs for ACP was not consistently available	✓ Culture of palliative care and openness to involve residents' wishes
Evidence‐based tools: STOP&WATCH	Lack of clarity regarding ward processes, such as feedback loops	✓ The introduction of a control mechanism for documentation increased accountability ✓ Mandated use of STOP&WATCH supported structured reporting and information sharing
ISBAR	Lacking confidence to use ISBAR with some physicians Licensed practical nurses displayed hesitancy in discussing «assessment» and «recommendation» as this is beyond their usual scope of practice	
Data driven quality improvement	Potential benefits and practical application of data were not well understood	

Another major impediment was the lack of confidence among those implementing the intervention elements, such as conducting ACP discussions or using the ISBAR tool with certain physicians. This lack of confidence was often due to unclear and impractical processes, like time constraints for ACP conversations or unclear procedures for using STOP&WATCH post‐completion by nurse assistants. Time restrictions also hindered structured interprofessional exchanges and ACP conversations.

Facilitators highlighted the importance of fostering an open culture of change within a participatory framework. Factors such as a shared vision between NH leadership and INTERCARE nurses, a participatory role development approach, a culture encouraging professional exchange (often informally), and a willingness to incorporate residents' preferences all contributed to a culture of openness. This enables mutual learning, inclusivity, and change.

## 4 Discussion

This process evaluation aimed to get a deeper understanding of how the INTERCARE intervention worked in practice to inform a larger‐scale implementation. We observed minor variations in the application of intervention elements across different NHs. Two primary mechanisms of change emerged from our evaluation: changing reasoning and enabling existing reasoning. Interview data indicated the limited application of comprehensive geriatric assessment and data‐driven quality improvement, highlighting central barriers to their implementation.

Our study helps to better understand how the INTERCARE intervention elements achieved a reduction in hospital transfers. As reported elsewhere, the INTERCARE model demonstrated effectiveness in significantly reducing unplanned transfers [10]. A follow‐up study showed that higher fidelity to the core elements ACP and evidence‐based tools, that is, ISBAR and STOP&WATCH, was significantly related to the reduction of unplanned transfers [12]. When examining moderating factors for fidelity, Guerbaai et al. [12] identified four key areas based on notes from meetings with NH leadership. First, contextual elements such as the presence of a responsible physician and the advanced experience of the INTERCARE nurse supported higher fidelity. Second, participants' positive responses to both the model itself and the role of the INTERCARE nurse contributed to sustained engagement. Third, internal strategies within nursing homes, including the identification of champions on the units, facilitated implementation. Finally, the commitment of nursing homes to delivering high‐quality care, particularly in relation to the INTERCARE nurse's role, further strengthened fidelity. These factors highlight the importance of contextual resources, organizational strategies, and stakeholder engagement in supporting successful implementation. This process evaluation builds on more extensive sources, adding to the NH leadership perspective. It corroborates former findings, that is, that the main core elements contributing to the model's effectiveness are the application of ACP, the use of evidence‐based tools, and the presence of an INTERCARE nurse and better interprofessional collaboration. In addition, this process evaluation explored the mechanism linking higher fidelity to these core elements with fewer unplanned transfers: INTERCARE's multifaceted approach targeted healthcare professionals' behaviors. The four core elements collectively influenced care workers' decision‐making, leading to improved attitudes, increased self‐confidence, quicker responses to resident changes, more thorough assessments, and enhanced communication, resulting in quicker GP responses to NH calls. Our findings contrast with a recent randomized controlled trial that focused on improving interprofessional collaboration with less favorable results in reducing NH resident transfers [16]. Although our overall design does not allow us to differentiate whether this combination of our intervention elements was more effective than applying them independently from each other, our study suggests a broader, multifaceted approach, as we used in INTERCARE, could be a key factor for its success. The increasing medical complexity of NH residents (e.g., multimorbidity, polypharmacy, frailty) and the growing complexity of nursing care might increase the risk of unplanned transfers. Accordingly, embedding trained INTERCARE nurses and evidence‐based tools for early detection and communication might also support matching care capacity to rising demands and minimize the risk that greater medical and care complexity translates into unplanned transfers.

The INTERCARE nurse emerged as a pivotal figure in changing the reasoning of care teams. As a crucial clinical contact, the INTERCARE nurse played a key role in facilitating more effective responses to acute situations, conducting comprehensive assessments, and improving interprofessional communication. This is in line with formerly published results that the INTERCARE nurses had high acceptability among team members due to their supporting role [11]. This transformative role aligns with social cognitive theory [17], which maintains that learning happens in a social context in an interplay of observation, imitation, and reinforcement. The INTERCARE nurse serves as a role model, demonstrating the expected behavior. Team members engage in vicarious learning by observing the behavior and imitating it, especially if they see a positive effect, be it for residents or the role model. By providing positive feedback, the INTERCARE nurse uses verbal persuasion to foster team members' confidence and self‐efficacy in actually performing the new behavior [18]. Their presence and continuous encouragement help team members to build mastery, working through failures, for sustained self‐efficacy. Their presence in difficult situations also helps to reduce stress, altering the affective state of team members to be able to better perform tasks. Overall, the INTERCARE nurse thus helps to build self‐efficacy of team members via all four principal sources of information exposed by Bandura [19]: vicarious learning, verbal persuasion, enactive mastery experiences, and altering physiological and affective states. In addition, via their modeling of clinical reasoning in, for example, case discussions, the INTERCARE nurse fosters a team‐based reasoning, helping the team to find common words for complex situations and build a collective confidence in handling situations. However, fostering self‐efficacy depends on organizational and system‐level influences.

The use of evidence‐based tools also supported these behavioral changes. Specifically, the STOP&WATCH tool facilitated early detection and response, while the ISBAR tool provided a structured approach to acute situations, fostering comprehensive assessments and organized communication processes. Improvements in communication and preparedness have been echoed by other studies [20, 21], with nurses reporting feeling better equipped to discuss resident issues [22].

Research consistently shows the positive impact of nurses in expanded roles within NHs, especially in promoting ACP and supporting decision‐making processes, thus reducing hospitalization risks [23, 24]. Despite some barriers, including the self‐efficacy of both INTERCARE nurses and care teams in initiating ACP conversations and limited GP involvement, the introduction of ACP raised staff's awareness of residents' preferences and helped in preparation for times when GPs were less available. However, further training and support for developing internal ACP processes were identified as needs. This is consistent with Gilissen et al., who identified training as a critical precondition for successful ACP implementation [25].

The INTERCARE intervention proved effective due to its multi‐pronged approach, addressing various care and decision‐making elements. Yet, recognizing and addressing barriers that hinder the full application of all intervention elements is crucial. While data‐driven quality improvement presents a promising avenue for enhancing care within NHs, our analysis showed that its adoption is a significant challenge. On the one hand, our minimal requirements for this core element required that NHs apply the PDCA cycle to a selected quality issue. Among all other minimal requirements in the context of the study, this might have been too burdensome, since NHs were not used to applying a full PDCA cycle in their quality improvement work. On the other hand, we noted a lack of understanding of how to effectively use the data. We observed an overall low level of data literacy with a high need to discuss quality data to understand their meaning, which is the basis for any quality improvement cycle. This might be linked to a rather low data maturity of NHs—as has been observed in a recent US study [26]—, with difficulties in accessing their own quality data, few monitoring systems in place, and little practice in applying data‐driven quality improvement [27]. Accordingly, the introduction of data‐driven quality improvement requires particular attention, beginning with strengthening data literacy and advancing data maturity. Switzerland only recently mandated electronic patient files in NHs and established quality indicators [28], introducing data‐driven quality improvement, while implementing an organizational change with a new care model, can be overwhelming. A recent study highlighted a decade‐long, multi‐level approach to implementing data‐driven quality improvement, involving membership rules, engagement monitoring, interactive assistance, and incentive structures [29]. This demonstrates the need for a phased, multi‐level approach, including educational initiatives, monitoring, interactive assistance, and staff motivation incentives. A corresponding initiative is currently ongoing in a Swiss national implementation program fostering data‐driven quality development in nursing homes [30]. Such an approach can help NHs to adopt data‐driven practices, enhancing care quality and reducing unplanned hospital transfers in the long run. For the planned scale‐up, support in this core element needs to focus on internal data structures and their use, building sustainable monitoring and benchmarking opportunities independent of the study team.

There were similar barriers to the adoption of CGA. Despite high adoption rates according to our minimal requirements, CGA was not perceived as contributing to reducing unplanned hospital transfers. This finding is consistent with our quantitative analysis of the relationship between the uptake rates of INTERCARE intervention elements and unplanned hospital transfers [12]. CGA can be defined as a “multidimensional, interdisciplinary diagnostic process focused on determining the medical, psychological, and functional capabilities of a frail elderly person to develop a coordinated and integrated plan for treatment and long‐term follow‐up” [31]. A realist review highlights the importance of a systematic approach to CGA, coupled with multidisciplinary team communication and coordinated care delivery for its effective use in NHs [32]. The operationalization of CGA used in INTERCARE currently focuses mainly on the aspect of systematic assessment of residents. To equip INTERCARE nurses for using CGA, they were taught to use different focus assessment instruments that go beyond the Resident Assessment Instrument‐Minimal Data Set (RAI‐MDS) (e.g., focusing on pain, malnutrition, or delirium) and to discuss with the NH leadership how teams could be better supported by introducing such structured instruments. While CGA has proven effective to reduce hospitalizations in community‐dwelling older adults [33] there is a lack of evidence on the effectiveness for NH residents. The continuous collection of data provides an overview of residents' bio‐psychosocial functioning, but the assessment, usually conducted by nurses, lacks a multi‐disciplinary perspective and is not sensitive to acute changes in residents' health conditions. Here, conducting an in‐depth assessment or timely referral to medical specialists might be more important measures to avoid hospitalizations, yet this was not included as a minimal requirement of the CGA element. Advanced clinical skills training, especially in early identification of deterioration and clinical management of conditions typically resulting in unplanned hospital transfers, was also highlighted as a core component in the study by O'Neill and colleagues [34], and was incorporated as a minimal requirement for the INTERCARE nurses and the interprofessional collaboration component. The limited recognition among participants of the link between CGA and unplanned transfers therefore reflects that CGA in INTERCARE primarily provided a structured framework for targeted assessments focused on specific clinical domains, such as pain or malnutrition, rather than on acute care needs. To achieve a stronger impact on reducing hospital transfers, INTERCARE nurses need to apply broader acute care skills. For the planned scale‐up of the INTERCARE intervention, revising the CGA element to focus more on early identification and management of deteriorations might be worthwhile.

This study has several limitations and limited generalizability due to its small sample and reliance on qualitative data from a single national context. By analyzing data from only five of the eleven participating sites, we may have overlooked additional insights. This might introduce a potential selection bias and affect the transferability of the findings to NHs with characteristics not represented in our sample. However, our maximum variation sampling strategy was designed to mitigate this potential gap. Additionally, this paper relies solely on qualitative data and does not integrate quantitative results from the main study. Nonetheless, our multi‐method triangulation design aimed to build theory rather than explore potential quantitative associations. A previous paper focusing on the fidelity of the core elements analyzed potential associations with their effect on unplanned hospitalizations by linking the respective data [12]. Another strength is its practical implications for refining insights into core components such as CGA and data‐driven quality improvement, which have been underused so far.

## 5 Conclusion

This process evaluation has provided valuable insights into the INTERCARE intervention, highlighting its strengths and areas for improvement. Effective interprofessional collaboration and the crucial role of the INTERCARE nurse emerged as key elements for success. The findings on ACP underscore its importance but also reveal the need for greater knowledge, additional training for care workers, and better collaboration with GPs. While comprehensive geriatric assessment and data‐driven quality improvement principles show promise, they remain challenging areas requiring better understanding and utilization within nursing homes. As the INTERCARE intervention moves towards potential scale‐up, these findings serve as essential pointers for refining its elements, enhancing its effectiveness in reducing unplanned hospitalizations, and improving residents' quality of life. Future research should address these challenges and investigate the sustainability of the intervention.

## Author Contributions

**Franziska Zúñiga:** conceptualization, data curation, formal analysis, funding acquisition, methodology, validation, writing – original draft, writing – review and editing. **Kornelia Kotkowski:** conceptualization, data curation, formal analysis, investigation, methodology, supervision, writing – original draft, writing – review and editing. **Raphaëlle Ashley Guerbaai:** conceptualization, formal analysis, investigation, methodology, writing – review and editing. **Michael Simon:** conceptualization, funding acquisition, investigation, methodology, validation, writing – review and editing. **Christine Serdaly:** methodology, validation, writing – review and editing. **Sabina De Geest:** funding acquisition, writing – review and editing. **Andreas Zeller:** funding acquisition, methodology, validation, writing – review and editing. **Reto W. Kressig:** funding acquisition, methodology, validation, writing – review and editing. **Nathalie I. H. Wellens:** methodology, validation, writing – review and editing. **Thekla Brunkert:** conceptualization, data curation, formal analysis, investigation, methodology, writing – original draft, writing – review and editing.

## Conflicts of Interest

The authors declare no conflicts of interest.

## Transparency Statement

The lead author, Franziska Zúñiga, affirms that this manuscript is an honest, accurate, and transparent account of the study being reported; that no important aspects of the study have been omitted; and that any discrepancies from the study as planned (and, if relevant, registered) have been explained.

## Supporting information


**Supporting Table S1:** Overview of Implementation Strategies. **Supporting Table S2:** Overview of themes and codes sorted by intervention element.

## Data Availability

The data that support the findings of this study are not available due to privacy or ethical restrictions. The interviewees did not consent in the passing on or further use of the data.
