# Supplementary material for: Understanding a Complex Intervention to Reduce Unplanned Hospitalizations From Nursing Homes: Process Evaluation of INTERCARE
Source: Health Sci Rep. 2026 Jan 11;9(1):e71748. doi: 10.1002/hsr2.71748 (PMC12790691; doi:10.1002/hsr2.71748)
Supplement: Supplementary file 1 — Supporting Table S1: Overview of Implementation Strategies. Supporting Table S2: Overview of themes and codes sorted by intervention element. [file HSR2-9-e71748-s001.docx]

**Supplemental material**

Understanding a complex intervention to reduce unplanned hospitalizations from nursing homes: Process evaluation of INTERCARE

**Table of content**

[Supplementary Table S1 2](#_Toc191233392)

[Supplementary Table S2 3](#_Toc191233393)

[Supplement Interview Guide G1 INTERCARE nurse 6 months 5](#_Toc191233394)

[Supplement Interview Guide G2 INTERCARE nurse 12 months 6](#_Toc191233395)

[Supplement Interview Guide G3 Focus group registered nurse 7](#_Toc191233396)

[Supplement Interview Guide G4 Focus group care workers 8](#_Toc191233397)

[Supplement Interview Guide G5 Interview physicians 9](#_Toc191233398)

## Supplementary Table S1

Overview of Implementation Strategies

| **Focus** | **Implementation strategy** | **Operationalization** |
| --- | --- | --- |
| Overall implementation support | Provide ongoing consultation | Bi-monthly implementation meetings (2h) between the nursing home leadership (incl. nursing directors, INTERCARE nurses) and the research group to support and reflect on the intervention elements' implementation, and to provide information. Structured meeting notes were collected to capture implementation processes and relevant experiences. |
|  |  | Bi-weekly implementation telephone calls (1h) between INTERCARE nurses and the study coordinator to support, reflect and address the implementation of the role, STOP&WATCH, and ISBAR and to identify problems. During the phone calls structured notes were collected to capture implementation processes and relevant experiences. |
| INTERCARE nurse | Conduct ongoing training | Provision of education and training for INTERCARE nurses (approximately 390 hours) throughout the study. Topics comprised: clinical skills (e.g., comprehensive geriatric assessment), leadership, communication, quality improvement and information about the intervention elements (e.g., STOP&WATCH, ISBAR). |
|  | Develop and distribute educational materials | Research group posted on an online educational and training platform different educational material, e.g., readings, videos were posted for the INTERCARE nurses to support implementation. |
|  | Make training dynamic | The training for INTERCARE nurses comprised blended learning with e-learning and in-person education approaches to support learning. |
| STOP&WATCH and ISBAR | Create new clinical teams | The INTERCARE nurses constituted a new member of the interprofessional care team and in this role their role is to facilitate the implementation of ISBAR and STOP&WATCH in the nursing home. They are responsible for planning, monitoring, evaluating the implementation in the nursing homes. |
|  | Develop and distribute educational materials | At the start of the implementation staff handouts, flyers, posters, PowerPoint presentations, and pocket versions of STOP&WATCH and ISBAR were distributed to the care workers. |
| Optional strategy | Identify and prepare champions | Nursing homes received implementation guidelines with the suggestion to appoint and train champions, i.e., local facilitators, on each unit to support the INTERCARE nurses in planning, monitoring, and evaluating both instruments' unit-level implementation. The INTERCARE nurse prepared the champions |

ISBAR: Introduction, Situation, Background, Assessment, Recommendation;

## Supplementary Table S2

Overview of themes and codes sorted by intervention element

| **INTERCARE nurse** | |
| --- | --- |
| Reduction in hospital admissions | More accurate assessment and evaluation |
|  | Improved communication with physicians |
|  | Faster response to health issues |
|  | Use of alternative treatment options that do not require hospitalization |
| Increased self-efficacy | Provision of support |
|  | Approachable contact person |
|  | Opportunity for joint reflection of situations |
| Relief for staff and care workers | Relief through opportunities to ask questions for residents, family, and nursing staff. |
|  | Departmental relief for staff and nursing management. |
|  | Reduction of stressful situations through support and relief. |
| Increased interprofessional exchange | Recognition of the importance of interprofessional exchanges. |
|  | Altered communication with physicians. |
| Improvement of quality of care | Enhanced communication among care workers for more specificity |
|  | Better differentiation of symptoms |
|  | Comprehensive examination of resident situations. |
|  | Increased team expertise. |
|  | Increased measurement of vital signs |
|  | Care workers asking more questions. |
|  | Improved documentation. |
|  | Care workers become more focused through systematic questioning. |
|  | More efficient reporting. |
|  | Structured orientation for new staff. |
| **Advance Care Planning** | |
| Clarified processes | Staff feels more secure with structured approach.  Development of a new staff attitude. |
| Clarified resident situations | Residents feel more secure. |
|  | Staff is better prepared for weekends |
|  | Staff is relieved |
|  | Use of PRN medication before contacting physicians. |
|  | Reduced deployment of mobile medical teams. |
| **Evidence- based tools** | |
| Stop & Watch | Improved documentation and more accurate transmission of information. |
|  | Reminders for professionals to evaluate situations. |
|  | More conscious perception of changes in resident situations and earlier response. |
|  | More efficient workflows (reporting, handovers) with fewer interruptions. |
|  | Increased appreciation for support staff through improved communication. |
| ISBAR | Improved preparation (visit efficiency/telephone). |
|  | More professional demeanor (structured, less forgetfulness, more confidence). |
|  | More mindful management of resident situations by trainees. |
|  | Gathering more information/vital signs for physicians. |
| Reflection tool | Early response |
|  | Identification of problematic areas needing optimization (e.g., missing vital signs, weekend preparation). |

## Supplement Interview Guide G1 INTERCARE nurse 6 months

Interview guide semi-structured interviews with INTERCARE nurses 6 after intervention start

*Unchecked translation with Microsoft Translator service.*

Evaluation

(Acceptance and feasibility)

**Research question:** How do INTERCARE nurses experience and shape the implementation of the core elements of the model?

**Interview Guide INTERCARE Nurses**

**Introduction**

- **Presentation of the topic**: My name is NAME. I am employed to conduct the interview with INTERCARE nurses as part of the INTERCARE study and today I would like to conduct an interview with you. Thank you very much for your willingness to participate in the interview. The interview will last about 60 minutes.
- **Procedure**: I will ask you open questions, you answer what comes to your mind or seems important. I have a guideline on which I have written down the topics that are important from my point of view. If you do not address the topics in this way, I will ask open questions.
- **Emphasize voluntariness:** Whenever you don't want to tell something, of course you don't have to. You can cancel the interview at any time if you don't feel comfortable doing so. Important: Of course, this has no disadvantages or consequences!
- **Confidentiality:** The interview is recorded so that the interview can then be written down and evaluated. If you would like to tell us something that is important to you, but that should not be included in the evaluation of the interviews, you are welcome to tell me. The contents of the interview will not be passed on to third parties, so your superiors or colleagues will never find out about the content.
- **Declaration of consent:** explain orally and present for signature if not already available (2 copies).
- **Questions:** Do you have any questions?
- **Is it okay if we start the interview and I turn on the tape recorder?**

About 6 months ago, a new care model was introduced in your home as part of the INTERCARE study. The model contains 6 core elements - show flyers and introduce the elements: Interprofessional cooperation, (you as) INTERCARE caregiver; multidimensional geriatric assessment; Forward-looking planning; evidence-based tools; and data-based quality development.

With this interview, we would like to better understand your experiences as an INTERCARE nurse after the first six months, i.e. from XX to today, in the implementation of the core elements of the model.

1. Can you start by telling me what your last/present working day as an INTERCARE nurse looked like, can you describe your day to me?

- What does your current working life look like?
- Was this more of a normal day or was it extraordinary?
- What happened that it was rather extraordinary?
- You mentioned that... why do you think it's important/unimportant?

General questions:

- Can you tell me more about it?
- What exactly did you do?

Implementation of the core elements

1. Collaboration / Acceptance – Feasibility

A core element of the model is interprofessional cooperation so that residents receive the best possible care. Now I would like to learn more about your role in the interprofessional team. Can you tell me how you have experienced the cooperation with other professional groups since the start of the model?

In-depth questions if it is difficult for interviewees to get in:

- Can you describe the last situation when you worked with the doctor?
- Do they have certain vessels for meetings with the doctor?
- How do you experience your cooperation with the doctor? How does he react to your role?
- What exactly is your role in working with...?
- What do you experience as difficult/easy?
- Why does it work/doesn't it work?
- Can you tell us something about how you felt?
- Has the collaboration changed over time?
- What do you think contributes to good cooperation?

How do you organise the cooperation with the Qualified Nursing Assistant I FaGe I Assistant Staff in everyday life?

- Can you describe a situation from this week or last week when you worked together?
- What do you experience as difficult/easy?
- Why does it work/doesn't it work?
- Has the collaboration changed over time?

General questions:

- What do you mean by that?
- Can you give me an example?
- How did you feel?
- Can you describe a situation from last week?

1. Multidimensional Geriatric Assessment/ Acceptance – Feasibility

If you think about the last two or three weeks, have you perhaps carried out an assessment, e.g. pain assessment, or fall assessment on a resident?

- Can you remember a similar situation?

If you are conducting assessments:

- How did you come to carry out the assessment?
- Can you describe the situation in more detail?
  - Who involved them?
  - Who was involved?
  - How is the team involved?
- What worked well / less well?
- What do you experience as easy to carry out (the assessment)?
- What helped you make it work?
- What do you find difficult to carry out (the assessment)?
- What significance does it have for the resident that you carry out an assessment ?
  - Does the assessment have an impact on the residents' situation?
  - Can you give an example?
  - Have you seen any changes in the resident situation?
- How do you combine your day-to-day activities with the performance of assessments?
- What other assessments can you implement in everyday life?

If she does not carry out assessments:

- How was the topic of "carrying out assessments" by INTERCARE caregivers addressed in your home?
- How do you see your role in carrying out assessments on a day-to-day basis?
- What do you think, can your everyday activities be combined with the implementation of the assessment?
- What would help you so that you can do it?
- What resources or support would you like to see?

1. ACP / Acceptance – Feasibility

Another core element is forward-looking planning, you have attended a training course on this and completed an e-learning course. In INTERCARE, you are not expected to have longer discussions with the residents about the topic. Perhaps you can remember the last situation when you talked to a resident about REA status, hospital admissions or other aspects of forward planning?

- How did you experience it?
- What do you experience as easy?
- What do you find particularly difficult?
- What helped make it work?
- What do you think is the added value for the residents in the conversation?
- What do you think such a conversation does to the resident, what goes down well?

In case she does not carry out the interviews

- How did you address this core element in the home? What is the role of INTERCARE nurses in this area?
- How do you feel about the idea of holding such conversations?
- What would help you, what would you wish for you to be able to do it?
- What resources or support would you like to see?
- What do you think is the added value for the residents in such a conversation?

General questions:

- Can you give me an example?
- Can you describe it in more detail?
- Can you describe a situation from last week?

1. Reflection Instrument / Acceptance – Feasibility

They are also involved in the implementation of evidence-based tools such as ISBAR, STOP and WATCH and reflection tools. I would now be interested to know how you experience the implementation. I would like to focus in particular on the implementation of the reflectiontool . Can you remember the last situation in which you filled out the reflection instrument?

- What happened in this situation?
- How did you experience the situation?
- How do you experience filling out the instrument?
- What do you find helpful and why?
- What do you experience as particularly difficult and why?
- How do you organize the reflections in the team?
- How do you experience the reflections in the team?
- How do the teams react to this?
- How do the doctors experience it?
- What significance does reflection have for the residents?
  - What do you mean by that?
  - Can you describe it?
- What do you think it will take for it to be used in the future?

General questions:

- Can you give me an example?

1. Data-based Quality Improvement Acceptance – Feasibility

Finally, I would like to talk about data-based quality improvement. A learning unit was also created in the curriculum on this topic. In general, it is about critically analyzing the RAI data, planning measures and then evaluating them. You will receive the SPC Charts on 4 quality indicators such as pain, malnutrition or hospital admissions every three months and then the benchmarking report for 6 months where you can compare yourself with other homes.

**(For Helena: I will let you know before the interview what the person / home has already received)**

I would like to know what your role is in everyday life in this context, i.e. in critical analysis, action planning and evaluation?

- If it has nothing to do with it:
- What tasks in your role are associated with projects or processes related to quality improvement?
- What was the last situation when you were involved in quality improvement?
- Can you describe the situation in more detail?
- What do you experience as easy?
- What do you find difficult?
- Why not?
- How do you see your role in the future in this area – quality improvement?
- What would you like to do in this area?

If no aspects of their role have to do with quality improvement:

- What do you think your role could be in this area?
- What would you wish for?

Extra question if finished earlier:

When you think about your everyday life and see the core elements, which element is the easiest / best to implement for you?

- What makes it easy to implement in everyday life?
- How do you experience it in everyday life?
- What exactly do you do in everyday life to make it work?
- Are there specific things that make it easier to implement?
- Are there specific things that make it difficult to implement?
- Can you give an example?

1. Would you like to add something that is still important to you?

Thank you for your time.

I'm going to turn off the tape now.

## Supplement Interview Guide G2 INTERCARE nurse 12 months

Interview guide semi-structured interviews with INTERCARE nurses 12 months after intervention start

*Unchecked translation with Microsoft Translator service.*

**INTERCARE Nurse Interview after 12 Months**

**Research question:** How do INTERCARE nurses experience the curriculum in relation to the implementation of their role?

- Response to the curriculum as an implementation strategy: how did the INTERCARE nurses experience the different elements of the curriculum and how did they use them to shape their role. What are the similarities and differences in use?
- Which elements of the curriculum would INTERCARE nurses change or adapt based on their own experience?

**Interview Guide INTERCARE Nurses**

**Introduction**

- **Presentation of the topic**: My name is NAME. I am employed to conduct the interview with INTERCARE nurses as part of the INTERCARE study and today I would like to conduct an interview with you. Thank you very much for your willingness to participate in the interview. The interview will last about 60 minutes.
- **Procedure**: I will ask you open questions, you answer what comes to your mind or seems important. I have a guideline on which I have written down the topics that are important from my point of view. If you do not address the topics in this way, I will ask open questions.
- **Emphasize voluntariness:** Whenever you don't want to tell something, of course you don't have to. You can cancel the interview at any time if you don't feel comfortable doing so. Important: Of course, this has no disadvantages or consequences!
- **Confidentiality:** The interview is recorded so that the interview can then be written down and evaluated. If you would like to tell us something that is important to you, but that should not be included in the evaluation of the interviews, you are welcome to tell me. The contents of the interview will not be passed on to third parties, so your superiors or colleagues will never find out about the content.
- **Declaration of consent:** explain orally and present for signature if not already available (2 copies).
- **Questions:** Do you have any questions?
- **Is it okay if we start the interview and I turn on the tape recorder?**

With this interview, we want to better understand your experience with the training you received within the project (see the table with the modules, see next page). We are particularly interested in two aspects: First, how did you experience the training and what are your experiences with it. In the second step, we would like to find out how you used the different parts of the training to shape your role as an INTERCARE nurse (or nursing expert/geriatric care expert/geriatric specialist, etc.)?

**Part 1**

**Reactions**: how do INTERCARE Nurses engage with the Curriculum? How relevant do they think it is? How satisfied are they with it?

1. First of all, I would like to ask you to tell us quite freely how you experience the training in relation to your role as an INTERCARE nurse? Just tell spontaneously what comes to mind.

Perceived need for training

- What did you imagine for the training?
- When you heard that there was an apprenticeship, what did it trigger in you, what were your thoughts about it?
- What is particularly relevant for you in the training?
- Were there things that were already very familiar to you based on your previous professional experience? What was new?

Experience of training

- How do you (personally) experience the training?
- What was so special / what makes it so special that it helped you?
- Which elements of the training have particularly motivated you?? e.g. to watch the e-learnings on ADAM or to participate in the expert meeting?
- Were there aspects of the training that were more of a hindrance for you?

**Learning** – What knowledge or skills have they learned, have there been changes in attitude, confidence or commitment as a result of the project?

Impact of training

If you could now tell us what significance or influence the training had for you personally:

- What can you personally get out of this training?
- What can you take from it for yourself, for your role?
- What did XY trigger for you?
- What did XY mean to you?
- You have now told you about XY (learning unit, meeting, person...) what was (not) helpful?
- Has anything changed in your way of working / in the understanding of your work with the training?

Now I would like to ask you how your role feels now:

- How do you rate yourself in your role today (compared to the beginning)?
- Where are you now?
- How am I supposed to understand it?

**Behavior** - Transfer of the content, usefulness? Do they utilize what they’ve learned from the program? / Collective action - actions to implement the role

(Maybe you could tell me what you think) How did this training influence the implementation of the core elements of the INTERCARE project and your role?

- What is the significance of education (relevant, not relevant) for a role like yours?
- What does the training mean for the implementation of your role (clinical activities, coaching, clinical leadership, promotion of the IPC)?
- What is the significance of the training for the implementation of the project? (Core elements e.g. CGA, ACP?)
- How did that come about?
- Do you have any explanation for this?
- Was that necessary / not necessary for you, what do you mean?
- What has become important to you during the project in terms of your activities?
- How have they changed?
- What helped you make these changes?
- What was the significance of the training in this?

**Results**

For you personally, what do you think is the impact of your role here in the company?

- Has anything changed in the company since your role was created?
- What is that?
- Are there any effects that are visible to others? (Residents, MA?)
- Are there effects that are important but not so visible to the outside world?
- Could you give a concrete example?
- Examples of results: better coordination, management of chronic diseases, changes in BW health status are recognized and evaluated more quickly?

**Improvements**

When you look back on the training now, is there anything you would add?

- Is there anything you think is unimportant?
- What would be the most important thing for you in your training?
- What would you change?

**Part 2**

How curriculum is offered – delivery of the curriculum

Now I would like to ask you in more detail how you experienced certain elements of the training.

1. It started with 5 whole days before the start of the project. Two days of clinical assessment with Morag Henry, 2 days of leadership and communication with Miriam Engelhardt and 1 day of geriatric assessment with Anja Ulrich.

- We saw in the evaluation that the days for the clinical assessment and the geriatric assessment performed worse than other topics. How can we improve them?

1. We have had a total of 8 half-day expert meetings. Four of them were with Miriam, two on the topic of Advance Care Planning with the team from Zurich, one on the topic of falls with Barbara Vogel and one on delirium with Wolfgang Hasemann. The expert meetings with Miriam Engelhardt were experienced as very helpful for the role as an INTERCARE nurse. The two afternoons on ACP performed worse.

- Could you first tell me what you would expect in a training on ACP so that you can implement it? What can we improve about this training?
- What led to the good rating of the meetings with Miriam? Do you have an explanation for that?

1. We have heard that the phone calls with Natalie (or with Kornelia for Christophorus) have a special meaning for many. What significance do the phone calls have for you?

- What has a special effect on you?
- Could you describe it?
- To what extent does it influence or influence your role at the time?
- How do you mean that it was (not) helpful?
- Are there any specific reasons for this?
- Did it have any impact on security?
- Why do you think it had no / an influence?
- What did you expect?
- What was particularly helpful for you, for your role?'

1. Maybe you could tell me briefly about the home accompaniment (with XY). (The content is confidential, it's about experiencing what was helpful, what wasn't etc.)

- Did the accompaniment meet your expectations?
- Was there anything that was particularly helpful for you, for your role?
- To what extent did it help you?
- What was particularly important for you in this accompaniment?

1. We have also made various e-learnings available on ADAM (show list?). Most of them concern clinical topics and were not experienced as very helpful for the role of INTERCARE nurses.

- What significance do these clinical topics have for you?
- Why do you think these have not been rated as helpful?
- Are there any topics that you find particularly helpful, topics that you have been missing so far?
- If there is enough time:
- In particular, two topics: hearing and visual impairment, and benchmarking were rated as not really helpful. What do you think, in the meantime the topics have been unlocked, maybe you have revised them and could say what you think?

If there is enough time:

This combination with the different learning methods, how was it for you?

- Would you weight it differently, e.g. offer certain topics more than expert meetings other than e-learning?
- How do you combine the different elements of the training with your work? What was easier to integrate, what was more difficult?
- How did you experience e-learning with ADAM?
- What do you think about the intensity of the training with the different meetings, the phone calls and the learning sessions on ADAM?

In your opinion, is there anything important that we have forgotten?

- What would you like to tell us if we continue to offer this training?
- What do you experience as difficult/easy?
- Why does it work/doesn't it work?
- Can you tell us something about how you felt?
- Has it changed over time?
- What do you think had the most influence on it?
- Can you describe a situation as you xx?
- What do you experience as difficult / easy?
- Why does it work/doesn't it work?
- What do you mean by that?
- Can you give me an example?
- What feelings did that trigger in you?
- What helped you the most to make it work?
- Are there any aspects that you find particularly difficult in the training?
- What does it mean to you ?
  - Does it have an impact on your security, skills?
  - Do you notice changes in yourself as a person? As an INTERCARE nurse?

Thank you for your time.

I'm going to turn off the tape now.

## Supplement Interview Guide G3 Focus group registered nurse

Guide for focus groups with registered nurses 6 months after intervention start

*Unchecked translation with Microsoft Translator service.*

Evaluation after 6 months (acceptance and feasibility)

**Focus Group Interview Guidelines for Professionals**

- **Presentation of the topic**: My name is NAME and I am doing my doctorate at the Institute of Nursing Science at the University of Basel. With me today is also NAME OF THE PERSON and she will support me today in conducting the focus group interview. We are both working on the INTERCARE project. As part of this project, a new, care-led care model was introduced here in the NAME nursing home, which is intended to **increase the quality of care**, **strengthen interprofessional cooperation** and avoid stressful **hospital admissions**. The model contains a total of six core elements (show flyer). Today we would like to talk about your experience and your experience of the model and two elements of the model. You work directly with the INTERCARE Nurse NAME and you may have come into contact with the STOP and WATCH instrument, so we are particularly interested in what you think about it.
- **Procedure**: I will ask you open questions, answer what comes to your mind or seems important. Ms. XX will present the main topics that occur today on the flipchart in the form of a "knowledge mapping". This means that it will present the results graphically. This will help us summarize the main points of today's conversation so that you can give us feedback on whether we have understood you correctly.
  There are no wrong or right answers, there are simply different opinions that you can express today. We are interested in all opinions.
- We have prepared blank name cards for everyone, could you please write your name on the card? You help us to have your name present. We want every opinion to be heard, so we ask that only one person speak at a time.
- **Emphasize voluntariness:** Whenever you don't want to tell something, of course you don't have to. You can cancel this focus group interview at any time if you don't feel comfortable doing so. Important: Of course, this has no disadvantages or consequences for you!
- **Confidentiality:** This focus group interview will be recorded so that the conversation can then be written down and evaluated. Only selected people from the research group at the institute have access to the interviews, their employer will not know what is discussed in this interview. If you would like to tell us something that is important to you, but that should not be included in the evaluation of the interviews, you are welcome to tell me.
- Thank you for participation and time commitment. The focus group interview will last about 60 to a maximum of 90 minutes. Does anyone have to leave earlier? Can beepers or telephones be handed in or switched off (if specialists)?
- **Declaration of consent:** Explain orally and present for signature if not already available (2 copies per person).
- **Questions:** Do you have any questions?

We would like to start with a short round of introductions: can you tell us your name and where you work?

- Thank you. Now I will start with the first question.
- **Is it okay if we start the interview and turn on the tape recorder?**

**Research question:** *How do employees experience and accept the role of IP and new instruments: ISBAR and STOP and WATCH, in their everyday lives?*

We would like to start with a short round of introductions: can you tell us your name and where you work?

- Thank you. Now I will start with the first question.

Acceptance model in general

1. They are all here because their home is involved in the INTERCARE project. Within the framework of INTERCARE, a new care model was launched in the MONTH / YEAR. INTERCARE includes 6 elements (show flyer – mention all elements). I would like to give you a few minutes to think about how you have experienced these innovations so far?

- How do you experience the elements of INTERCARE in your everyday life?
- Has anything changed in your everyday life since you started with INTERCARE?
- What are the positive / negative aspects of INTERCARE in everyday life?
- What causes the positive/negative thoughts?
- What do you think about INTERCARE works particularly well/badly in everyday life, what are your thoughts on this?
- How is your work influenced by INTERCARE, what has changed?
- What do other employees think about INTERCARE?

Summary: The second person makes a summary of the conversation for about two minutes - the participants are asked what comments or adjustments you have.

Acceptance, feasibility of the IP role

Acceptance, feasibility of the IP role (always ask for the name when the people are telling)

1. Tell us about your cooperation with the INTERCARE nurse or the NAME of the person or name of the role used in the home (e.g. nursing expert, geriatric care expert – use)?. The role was newly introduced here in the home **OR** the role already existed before the project, but in the context of the project it was given new, additional tasks. When you think about this role now, how do you experience it in everyday life?

- What do you think about this role?
- How do you experience working with NAME on a day-to-day basis?
  - What do you find easy/difficult in working with NAME?
  - To what extent does this role influence your everyday life?
  - Do they find the role helpful/unhelpful?
  - Are there any positive/negative aspects of the role of NAME?
- What was the last situation when you worked with NAME?
  - Can you describe this situation?
  - Why did you bring her / him?
  - Who got her/him?
  - What worked well/not so well?
  - Is there any other situation where you work with the NAME?
- What other support do you receive from NAME in your everyday life?
- In which situations would you still like support?
- If the role is new in the home: Has anything changed in the course of time, since the beginning of the project in the cooperation with NAME?
- If the role already existed, has anything changed in the cooperation with NAME since the start of the project compared to the past?

Summary: The second person makes a summary of the conversation for about two minutes - the participants are asked what comments or adjustments you have.

Acceptance / Feasibility Instruments

3. Two instruments have been introduced as part of the model: STOP and WATCH, ISBAR (Show Instruments).

4.1 ISBAR

- Have you already dealt with the ISBAR instrument in the last few months?
  - If so, can you remember the last time you used ISBAR:
    - In what situation did you use the instrument?
    - Can you tell us more about this situation?
    - Can you describe it in more detail?
    - What were the consequences?
    - What do you think would have happened if you hadn't used ISBAR?
  - How does the instrument affect communication in everyday life?
- Do you see positive / negative aspects in the use of the ISBAR?
- If so, which ones? Can you describe them in more detail?
- Have there been any changes in communication with doctors since you started using ISBAR?
- How do you experience the use of ISBAR in your department?
- How does ISBAR work in your department / How is the instrument used?
- What makes it difficult/easy to integrate ISBAR into everyday life?
- Do you need more support in using it? (if so, what exactly?)

Summary: The second person makes a summary of the conversation for about two minutes - the participants are asked what comments or adjustments you have.

4. STOP and WATCH

Now I would like to talk about the STOP and WATCH. The STOP and WATCH is a structured and simple communication tool that helps the staff who are in regular contact with the residents to recognize changes in their general state of health, e.g. someone suddenly eats less or has to go to the toilet more often than usual, and to report it to the responsible nurse (show instrument)

- How do you experience the use of the STOP and WATCH in your department?
- What makes it difficult/easy to integrate the instrument into everyday life?
- What does it take to make it work better?
- What do they do when you receive a completed STOP and WATCH?
- How do you decide on the best course of action, whether to bring someone into the situation?

Summary: The second person makes a summary of the conversation for about two minutes - the participants are asked what comments or adjustments they have.

1. If you could just change something about INTERCARE now, about everything that has been introduced, what would you do?

- What would you recommend?
- What would they do differently?
- What should stay the same?

Summary: The second person makes a summary of the conversation for about two minutes - the participants are asked what comments or adjustments you have.

At the end:

1. We talked about a lot of things today. What is the most important point you would like to give us? Would you like to add something that is still important to you?

Thank you for your time.

**What's next:** Transcription and analysis, summary of focus groups across all participating homes. Summary report on the results of all the homes that the homes receive. There are no names in it and no statements can be assigned to individual persons. The homes can then decide how to proceed with the results.

Extra questions for other elements:

**ICP**

As part of the project, we also want to improve interprofessional cooperation. How do you experience the cooperation with the other professional groups?

- Has cooperation with other professional groups changed recently?
- Who are they most in contact with?
- Has cooperation with other professional groups changed recently?
- Do you still see areas where cooperation can be improved?

**CGA**

I would like to ask if you have experienced any changes in the use of assessments in the last 6 months?

- Have new assessments been introduced? (e.g. pain assessment, or fall assessment)
- Has anyone talked to you about possible assessments?
- Have they received any training on this?
- Do they have a specific role in the use of assessments?

**ACP**

Yes, one of the goals of the project is to reduce hospital instructions by consciously designing forward-looking planning. This means that it is discussed with the residents at an early stage what wishes and concerns they have if acute situations occur or their general condition deteriorates. Have they perhaps come into contact with this element, have they heard about it?

- What do you think about such planning in advance for residents?
- Are you involved in this planning and clarification of the wishes?
- Are the residents' wishes discussed here in the home at an early stage with regard to hospital admission or REA status?
- Is it quite possible for you to find out what wishes residents have with regard to these topics, e.g. in the documentation?

**Reflexionsinstrument**

There is another instrument – a reflection instrument. After each unplanned hospital admission, the instrument should be filled out and analyzed with the team. So that the INTERCARE nurses and we can analyze them, and look at what the most common reasons for these hospital admissions are. Have they already come into contact with the reflection instrument?

- Have you ever been involved in a reflection on a hospital admission?
- How did you experience it?
- What was easy/difficult?
- What did you take away from the reflection?

**Data-based quality development**

As part of the project, we regularly collect data. This data is discussed with the INTERCARE nurse and other managers. Have they heard anything about it?

- Have you heard that we collect and analyze data?
- Does that have any significance for them in everyday life?
- Has anyone presented and discussed the data?
- Would you like to know more about the results of the data collection?

**Follow-up**

– Special features of the survey situation and personal impression of the interviewees according to

Record the focus group interview on the appropriate protocol, make field notes:

1. My impression of the other person was...
2. I have experienced the focus group interview....
3. Central places that caught my eye...

Contextual information about the subjects that are important:

1. Dialect
2. Physical expression, emotions

Material Interview

– 2 recording devices (spare device!)

– possibly power cable/busbar or spare batteries

– Copies of the interview follow-up

## Supplement Interview Guide G4 Focus group care workers

Guide for focus groups with care workers 6 months after intervention start

*Unchecked translation with Microsoft Translator service.*

Evaluation after 6 months (acceptance and feasibility)

**Focus Group Interview Guidelines for Assistants**

- **Presentation of the topic**: My name is NAME and I am doing my doctorate at the Institute of Nursing Science at the University of Basel. With me today is also NAME OF THE PERSON and she will support me today in conducting the focus group interview. We are both working on the INTERCARE project. As part of this project, a new, care-led care model was introduced here in the NAME nursing home, which is intended to **increase the quality of care**, **strengthen interprofessional cooperation** and avoid stressful **hospital admissions**. The model contains a total of six core elements (show flyer). Today we would like to talk about your experience and your experience of the model and two elements of the model. You work directly with the INTERCARE Nurse NAME and you may have come into contact with the STOP and WATCH instrument, so we are particularly interested in what you think about it.
- **Procedure**: I will ask you open questions, answer what comes to your mind or seems important. Ms. XX will present the main topics that occur today on the flipchart in the form of a "knowledge mapping". This means that it will present the results graphically. This will help us summarize the main points of today's conversation so that you can give us feedback on whether we have understood you correctly.
  There are no wrong or right answers, there are simply different opinions that you can express today. We are interested in all opinions.
- We have prepared blank name cards for everyone, could you please write your name on the card? You help us to have your name present. We want every opinion to be heard, so we ask that only one person speak at a time.
- **Emphasize voluntariness:** Whenever you don't want to tell something, of course you don't have to. You can cancel this focus group interview at any time if you don't feel comfortable doing so. Important: Of course, this has no disadvantages or consequences for you!
- **Confidentiality:** This focus group interview will be recorded so that the conversation can then be written down and evaluated. Only selected people from the research group at the institute have access to the interviews, their employer and the INTERCARE nurse will not know what is discussed in this interview. If you would like to tell us something that is important to you, but that should not be included in the evaluation of the interviews, you are welcome to tell me.
- Thank you for participation and time commitment. The focus group interview will last about 60 to a maximum of 90 minutes. Does anyone have to leave earlier? Can beepers or telephones be handed in or switched off?
- **Declaration of consent:** Explain orally and present for signature if not already available (2 copies per person).
- **Questions:** Do you have any questions?

We would like to start with a short round of introductions: can you tell us your name and where you work?

- Thank you. Now I will start with the first question.
- **Is it okay if we start the interview and turn on the tape recorder?**

**Research question:** *How do employees experience and accept the role of IP and the new STOP and WATCH instrument in their everyday lives?*

Acceptance model in general

1. They are all here because their home is involved in the INTERCARE project. Within the framework of INTERCARE, a new care model was launched in the MONTH / YEAR. INTERCARE includes 6 elements (show flyer – mention all elements). I would like to give you a few minutes to think about how you experience the time since XX MONTH?

- Do you see any positive/negative aspects of INTERCARE in everyday life?
- How do these positive / negative aspects come about, what contributes to them?
- How do your colleagues see it?
- What do your colleagues think about INTERCARE works particularly well/poorly in everyday life?
- What is your impression of what has been expected of you since the beginning of the project?
- What do you think about these expectations?
- What do your colleagues say about this?

Summary: The second person makes a summary of the conversation for about two minutes - the participants are asked what comments or adjustments you have.

1. I showed you the flyer. Here are the 6 elements that we as a research group would like to implement here in the home. You have already mentioned an item/a few items above. What other elements from this flyer do you recognize or work with?

- Then go to specific questions (STOP and WATCH or IP)
- How do you experience the elements/element in your everyday life?
- What do your colleagues in the departments think about the element(s)?

Acceptance, feasibility of the IP role (always ask for the name of the IP when people are talking)

1. We would like to hear about your cooperation with the INTERCARE nurse (or use the name of the person or the name of the role used in the home, e.g. nursing expert, geriatric care expert)? The role was newly introduced here in the home **OR** the role already existed before the project, but in the context of the project it was given new, additional tasks. When you think about this role now, how do you experience it in everyday life?

- What did you think about this role at the beginning?
- What did you hear from your colleagues about this role at the beginning? How did you talk about it, what were the expectations?
- How do you or your colleagues experience working with NAME in everyday life today?
  - What do you or your colleagues think it takes for the cooperation to work well?
  - What do you or your colleagues experience as easy/difficult in working with NAME?
  - What does this role bring you, is it helpful or unhelpful?
  - Are there any positive/negative aspects of NAME's role?
- What was the last situation when you worked with NAME?
  - Can you describe this situation?
  - Why did you bring her / him?
  - Who got her/him?
  - What worked well/not so well?
  - Is there any other situation where you work with NAME?
- What other support do you receive from NAME in your everyday life?
- In which situations would you still like support?

Summary: The second person makes a summary of the conversation for about two minutes - the participants are asked what comments or adjustments you have.

Acceptance / Feasibility Instruments

1. You mentioned the STOP and WATCH (STOP and WATCH - show instrument). STOP and WATCH is a structured and simple communication tool that helps staff who are in regular contact with residents to identify changes in their general health, e.g. if someone suddenly eats less or needs to go to the toilet more often than usual, and report it to the responsible nurse.

- What do you / your colleagues think about the instrument?
- What are positive/negative aspects in everyday life?
- What makes it difficult/easy to integrate the instrument into everyday life?
- What do you think is expected of you when using the STOP and WATCH?
- What does it take for the instrument to be used in your department?
  - What do your colleagues want to use the instrument in everyday life?
  - Is there anything that should change in dealing with STOP and WATCH? If so, what?
- How is the cooperation with other nurses or professionals now that they have been using the instrument?
  - Is there something for them or colleagues that is different now than before?
- Can you describe the last situation when you used the STOP and WATCH?
  - Can you give us an example when you used STOP and WATCH?
  - Can you describe it in more detail?
  - What led you to fill out the instrument?
  - To whom did you give the STOP and WATCH?
  - What happened after that, what happened to the resident?

Summary: The second person makes a summary of the conversation for about two minutes - the participants are asked what comments or adjustments you have.

1. If you could just change something about INTERCARE now, about everything that has been introduced, what would you do?

- What does it take for such a project to be well accepted?
- What is important for you or your colleagues?
- What would your colleagues wish/expect?
- What would you or your colleagues do differently?
- What should stay the same?

Summary: The second person makes a summary of the conversation for about two minutes - the participants are asked what comments or adjustments you have.

At the end:

1. We talked about a lot of things today. What is the most important point you would like to give us? Would you like to add something that is still important to you?

Thank you for your time.

**What's next:** Transcription and analysis, summary of focus groups across all participating homes. Summary report on the results of all the homes that the homes receive. There are no names in it and no statements can be assigned to individual persons. The homes can then decide how to proceed with the results.

Extra questions for other elements:

**ICP**

As part of the project, we also want to improve interprofessional cooperation. How do you experience the cooperation with the other professional groups?

- Who are you in contact with the most?
- Who else would you like to work with?
- Do you still see areas where cooperation could be improved?

**CGA**

I would like to ask if you have experienced any changes in terms of assessing resident situations in the last 6 months or in terms of assessment tools?

- Have new assessments been introduced? (e.g. pain assessment or fall assessment)
- Has anyone talked to you about possible assessments?
- Have you received any training on this?
- Do you have a specific role in the use of assessments?

**ACP**

One goal of the project is to reduce hospital instructions by consciously designing forward-looking planning. This means that it is discussed with the residents at an early stage what wishes and concerns they have if acute situations occur or their general condition deteriorates, e.g. with regard to hospital admission or REA status. Have you perhaps come into contact with this element, have you heard of it?

- What do you think about such planning in advance for residents?
- Are you involved in this planning and clarification of the wishes?
- Are the residents' wishes discussed here in the home at an early stage with regard to hospital admission or REA status?
- Is it quite possible for you to find out what wishes residents have with regard to these topics, e.g. in the documentation?

**Follow-up**

– Special features of the survey situation and personal impression of the interviewees according to

Record the focus group interview on the appropriate protocol, make field notes:

1. My impression of the other person was...
2. I have experienced the focus group interview....
3. Central places that caught my eye...

Contextual information about the subjects that are important:

1. Dialect
2. Physical expression, emotions

Material Interview

– 2 recording devices (spare device!)

– possibly power cable/busbar or spare batteries

– Copies of the interview follow-up

## Supplement Interview Guide G5 Interview physicians

Interview guide for interviews with physicians 6-9 months after intervention start

*Unchecked translation with Microsoft Translator service.*

Evaluation after 6 months

(Acceptance)

**Interview Guide Doctor**

**Introduction**

- **Presentation of the topic**: My name is NAME. I am the project coordinator in the INTERCARE study. They look after residents in the NAME nursing home, which participates in INTERCARE. INTERCARE is carried out by the Institute of Nursing Science at the University of Basel together with the Institute of Family Medicine and the Center for Geriatrics and Rehabilitation at the Felix-Platter Hospital. Its aim is to reduce stressful hospital admissions from nursing homes and to increase the quality of care. A special focus of the project is interprofessional cooperation.
- Thank you very much for agreeing to participate in this interview. I would like to talk to you about your experience of interprofessional cooperation with the NAME nursing home and in particular about your experience of communication with INTERCARE nurses NAME and the nurses in the NAME nursing home. The interview will last about 30 minutes.
- **Procedure**: I will ask you various open questions, answer what comes to your mind or what seems important. I have a guideline on which I have noted the topics that are important from my point of view. If you do not address certain topics on your own, I will ask.
- **Emphasize voluntariness:** Whenever you don't want to tell something, of course you don't have to. You can cancel the interview at any time without giving a reason and without any disadvantages or consequences for you!
- **Confidentiality:** The interview is recorded so that the interview can then be written down and evaluated. If you would like to tell us something that is important to you, but that should not be included in the evaluation of the interviews, you are welcome to tell me.
- **Declaration of consent:**

**If there is a declaration of consent:** Thank you for the written declaration of consent. It has been received by me.

**If there is no declaration of consent:** I have not received the written declaration of consent from you. Can we now record the informed consent form with the tape? Yes, do you agree with the interview, the procedure and the recording of the conversation?

- **Questions:** Do you have any questions?.
- **Is it okay if we start with the interview? I will now start recording the conversation.**

*Research question: How do doctors experience the cooperation with the home, the interprofessional communication with the nurses and the INTERCARE nurses and the role of the INTERCARE nurses in everyday life?*

In the INTERCARE study, we have implemented a new model that aims to increase the quality of care and strengthen interprofessional cooperation. One element of the model is the introduction of an INTERCARE nurse. This is a qualified nurse with advanced knowledge in the geriatric field. In addition, we are introducing the ISBAR instrument for structured communication with doctors.

1. The home NAME started with the model about 6 months ago (**name month**). Have you noticed a change in the cooperation with the home?

- How have you perceived the cooperation with the home in the last 6 months?
- Does something else work, what, how is it?
- What works the same?
- Has anything improved/deteriorated?

**Always: Ah so, hmmm, aha, I understand, yes, really, yhy...**

- Have you noticed any impact on your everyday work in the last 6 months?

1. Now I would like to talk specifically about communication: Can you tell me what your experience is in communicating with the **HEIM NAME** in relation to resident care?

- Think of the last situation in which communication with the NAME home went very well. Can you briefly describe the situation?
  - Who was involved in the situation?
  - Was the situation different than usual?
  - How did you experience the information transfer yourself?
  - Have you received all the relevant information?
  - Was the information passed on differently than otherwise?
  - What did you particularly like about communication?
  - What do you think could be the reason for this?
- Think about the last situation when communication with the NAME home didn't go so well, where it was difficult for you. Can you briefly describe the situation?
  - Who was involved in the situation?
  - What was particularly difficult for you to communicate with?
  - Was the communication different than usual?
  - How did you experience the passing on of information, was it different than usual?
  - What particularly bothered you in communication?
  - What do you think could be the reason for this?
- Is there a difference in how you experience communication about resident care, depending on who you are in contact with? (if necessary, give the examples: e.g. with diplomas or FaGe, assistants, INTERCARE nurses - NAME)
  - What do you think is the reason for the differences?
  - How do you notice the differences?
  - What do you think hinders effective communication between nurses and doctors?
  - What do you think is particularly helpful in communication between nurses and doctors? / What could improve communication?
  - What tip could you give caregivers (in terms of communication), what could the caregiver do to improve communication?

1. Now I would like to ask you specifically about the cooperation with the INTERCARE nurse NAME. Can you tell me how you experience the cooperation with INTERCARE Pflegede Name in everyday life?

- What was the last situation when you had contact with the INTERCARE nurse NAME ?
- How did you experience the INTERCARE nurse **NAME** in this situation?
- Can you give a concrete example of what is going well and where there is room for improvement?
- Are there any aspects that you have demanded in the cooperation?
- Are there aspects that you feel are beneficial?
- What helped you to have a good collaboration?
- Are there aspects of the collaboration that have enriched you?
- Can you give an example?
- Do you feel that...
- You mentioned earlier that...

1. **Extra**

If the improvement of communication in terms of structure was not mentioned, then specifically after ISBAR questions: I said at the beginning that we have introduced an ISBAR instrument for structured communication with doctors. I would like to ask you if you notice anything that has changed in the way information is passed on, e.g. during rounds or on the phone?

- Can you give an example ?

General questions:

- Why yes/why not?
- Why / for what reason?
- What do you mean exactly?
- Can you tell me a little more / are there any other things?
- Can you give me a concrete example?
- Can you describe a situation?

1. Would you like to add something else that you find important?

Thank you very much for your time.

**Follow-up**

- Record the special features of the survey situation and personal impression of the interviewee on the appropriate protocol after the interview, field notes:

1. My impression of the other person was...
2. I experienced the interview ....
3. Central places that caught my eye...

Contextual information about the subjects that are important:

1. Dialect

**Material Interview**

– Connecting recording devices to the phone (replacement device!)

– Possibly power cable/busbar or spare batteries

– Copies of the interview
